# Molecular Engineering of Quinone-Based Nickel Complexes and Polymers for All-Organic Li-Ion Batteries

**DOI:** 10.3390/molecules27206805

**Published:** 2022-10-11

**Authors:** Yanislav Danchovski, Hristo Rasheev, Radostina Stoyanova, Alia Tadjer

**Affiliations:** 1 Faculty of Chemistry and Pharmacy, University of Sofia, 1164 Sofia, Bulgaria; 2 Institute of General and Inorganic Chemistry, Bulgarian Academy of Sciences, 1113 Sofia, Bulgaria

**Keywords:** organic electrode materials, coordination polymers, DFT, periodic calculations, energy storage, redox potential, high capacity

## Abstract

All-organic Li-ion batteries appear to be a sustainable and safer alternative to the currently-used Li-ion batteries but their application is still limited due to the lack of organic compounds with high redox potentials toward Li^+^/Li^0^. Herein, we report a computational design of nickel complexes and coordination polymers that have redox potentials spanning the full voltage range: from the highest, 4.7 V, to the lowest, 0.4 V. The complexes and polymers are modeled by binding low- and high-oxidized Ni ions (i.e., Ni(II) and Ni(IV)) to redox-active para-benzoquinone molecules substituted with carboxyl- and cyano-groups. It is found that both the nickel ions and the quinone-derived ligands are redox-active upon lithiation. The type of Ni coordination also has a bearing on the redox potentials. By combining the complex of Ni(IV) with 2-carboxylato-5-cyano-1,4-benzoquinones as a cathode and Ni(II)-2,5-dicarboxylato-3,6-dicyano-1,4-benzoquinone coordination polymer as an anode, all-organic Li-ion batteries could be assembled, operating at an average voltage exceeding 3.0 V and delivering a capacity of more than 300 mAh/g.

## 1. Introduction

In synchrony with the global priority for a massive installation of sustainable energy storage technologies, it has been recognized that organic Li-ion batteries are becoming the incontestable winner in the race with the widely-spread current Li-ion batteries, where inorganic materials are mainly employed [1,2]. This is a consequence of the inherent properties of organic materials such as a diversity of structures and compositions and their tunable modeling, low-environmental footprint, etc. [3,4,5,6]. Both inorganic and organic materials store energy through redox electrochemical reactions, but the mechanisms are different [1,2]. For the inorganic materials, the electrochemical reaction proceeds thanks to the redox properties of transition metal ions, which compensate for the charge of Li^+^ insertion and extraction [7]. Only in certain cases of Li-rich layered oxides is the oxygen suspected in an oxidation state change as well. [8]. For the organic electrodes, the redox functional groups or/and metal centers participate in the electrochemical reaction and the charge compensation is achieved either by cations (i.e., Li^+^, Na^+^, Mg^2+^ charge carriers) or by anions from the electrolyte [9]. Although there are three important classes of inorganic electrodes [7], organic materials constitute an enormous database of electrodes, including conjugated carboxylates, amines, sulfonamides and their metal salts, carbonyl compounds, nitro-aromatics, organic radicals, conductive polymers, Hückel-stabilized Schiff bases, nitrogen-redox azo compounds and N-substituted salts of viologen derivatives [3,4,5,6,10,11]. Given that the organic electrodes cover a large range of potentials and specific capacities, all-organic Li-ion batteries can be successfully constructed, thus becoming a priority option for further development.

Inside the organic electrode database, metal-organic complexes (MOCs) can be outlined since they combine the advantages of the organic electrode materials with, for example, the excellent electrochemical properties and ordered structure of the inorganic ones [12,13]. In addition, with appropriate functionalization, MOCs with polyfunctional organic ligands can be assembled into 1D-, 2D-, or 3D-coordination polymers. Depending on the redox mechanism, MOCs can be divided into three groups: those with ‘redox-innocent’ ligands where only the metal changes its oxidation state; complexes where only the ligands are redox-active; and complexes in which both the metal and the ligands exchange electrons [14,15,16,17,18,19,20,21,22,23,24]. In this respect, a good choice for redox-active metal centers with a variety of oxidation states is the transition metals, particularly the first-row d-elements, as they are cheaper and lighter than their next-periods analogs. Among the first uses of MOCs as electrode materials was the reported in 2009 vanadium acetylacetonate complex acting as a symmetrical electrode by changing the metal oxidation states of vanadium in the V(II)/V(III) and V(III)/V(IV) pairs [25]. Besides the development of acetylacetonate-based complexes [26], different ligands were also implemented such as bipyridine-based [27] and terpyridine-like [28] in which the ligands support the redox process as well. Like vanadium, nickel offers a diversity of oxidation states and serves as a coordination center in MOCs providing an excellent storage performance [29,30]. For example, the Ni-thio-crown complex operating through Ni(I)/Ni(II) and Ni(II)/Ni(III) pairs retains its structure during the electrochemical reaction, which enables the use of this complex as an anode and cathode in an all-organic ion cell [31].

Among the organic materials with redox-active organic ligands, quinones are emerging as the primary choice for high-energy electrodes due to their two-electron redox reaction between Li and the carbonyl groups, the relatively small molecule (contributing to a high theoretical capacity) of benzoquinone (BQ) and simple modification of the structure [32,33,34,35,36]. From the two BQ isomers, ortho- and para-, ortho-BQ exhibits a somewhat higher potential than the para-analog, as well as a higher difference between the potentials of the first and second redox reaction [9,37]. Due to the latter, para-BQ is more interesting for practical applications. The potential of quinones can further be fitted to the sought application through the addition of electron-withdrawing groups such as carboxyl- (ester), cyano- [38], etc., groups. Quinone ligands, in particular, could be part of more elaborate structures, such as MOCs, coordination or functionalized polymers [39,40,41], and metal-organic frameworks [42,43]. The use of polymers with quinone monomers [44] is a recent idea that did not deliver the desired results at first, due to the structural instability of the polymers. Despite the discouraging outcomes of the metal-free polymer, coordination quinone polymers are reported to exhibit the desired stability and good electrochemical parameters [45]. The coordination polymer constructed by Xiang et al. uses the Li-O bonds as polymer linkers which eventually lead to decomposition. A recently proposed quinone-based 1D-metal-organic polymer of Cu experimentally demonstrates the success of the stated strategy—the combination of redox-active ligands and coordination centers participating jointly in the redox processes [46]. However, these materials need a more extensive exploration of their properties by diversification of the metal ions and the ligands. The primary results are promising—the polymers are stable, the Li insertion is easily possible, and the initial capacities are good. A further strategy for enhanced conductivity and cycling stability of these materials is strongly needed.

Irrespective of the extensive research on organic electrode materials, the practical application of all-organic Li-ion batteries is still limited due to the lack of organic compounds with high redox potentials toward lithium [5,47]. Among cathode materials, the highest potentials at 3.2–3.5 V have been established for conjugated sulfonamides and their Li and Mg salts [48], while, for anode materials, conjugated carboxylates, azo compounds and viologen-derived salts display low potentials (i.e., between 0.5 and 0.7 V). The battery assembled from the above cathodes and anodes will be characterized with an operating voltage lower than 3.0 V, which is below that of the inorganic Li-ion batteries (i.e., above 3.9 V using layered oxides as cathodes [7] and graphite as the anode). That is why the state-of-the-art studies are mainly directed at identifying organic compounds as electrodes that feature a potential difference of more than 3.0 V. 

**Figure 1:**
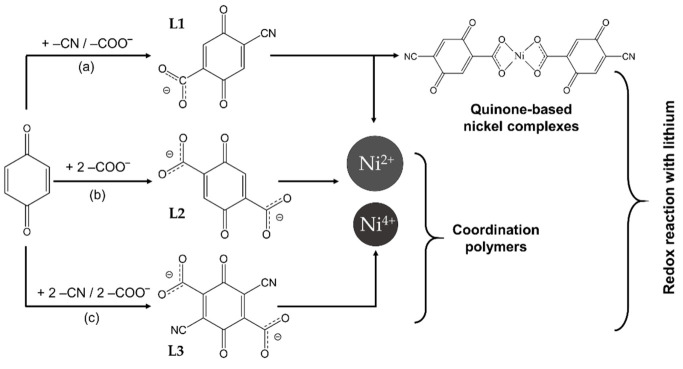
Schematic representation of the designed constructs and the study objectives: 1,4-benzoquinone functionalized with (**a**) a –COO^−^ and a -C≡N group (L1) to be used as a ligand in complexes with Ni(II) and Ni(IV); (**b**) two –COO^−^ groups (L2) or (**c**) two –COO^−^ and two -C≡N groups (L3), to be used as ligands in coordination polymers with Ni(II) and Ni(IV); the assemblies comprising Ni(IV) are neutralized by Cl^−^ ions.

The aim of this study is threefold: first, to design 0D and 1D redox-active constructs using the same metal centers and analogous ligands; second, to gain insight into the redox reactions of the designed coordination complexes and polymers with Li atoms; and third, to analyze their redox potentials and specific capacities aiming to propose the assembly of an all-organic Li-ion battery. As metal centers, nickel ions in two oxidation states (i.e., Ni(II) and Ni(IV)) are selected since these ions usually enable the achievement of high potentials in inorganic electrode materials. To find redox-active ligands with weights as low as possible (to increase the capacity and the gravimetric energy density), we focused on BQ-based derivatives, since their redox activity is well documented [38]. Thus, the chosen ligands are para-BQ molecules modified by adding –COO^−^ and -C≡N groups ensuring the coordination of Ni ions and targeting an enhancement of the redox potential. The construction of quinone-based Ni complexes and coordination polymers is presented in Figure 1. To understand the mechanism of the redox reactions of the complexes and polymers with Li, the corresponding ligands are used as references. The calculations of the geometry and electronic structure of lithiated complexes and polymers, as well as of the corresponding redox potentials, are carried out by means of DFT calculations. 

## 2. Results and Discussion

### 2.1. Transition Metal Complexes

The redox complexes are built from Ni ions as metal centers and 2-carboxylato-5-cyano-1,4-benzoquinone (L1) as ligands (Figure 1a). The choice of 1,4-BQ over the ortho-analog is based on the following reasons: (i) the redox potential profile of para-BQ is more consistent, as the difference between the redox potentials of the two steps of reduction is closer (i.e., 2.66 and 2.45 V compared to 3.67 and 2.50 V for ortho-BQ [37]), which is an advantage from a practical point of view; (ii) due to higher symmetry, the para-BQ ligand offers a smaller number of positional isomers upon further functionalization; (iii) reduced repulsion of the spatially separated Li^+^ ions obtained after reduction. For the para-quinone molecule, the introduction at selected positions of electron-withdrawing groups, such as N≡C-groups and carboxyl- (ester) groups, leads to the enhancement of the redox potentials and ensures effective complexation with the Ni ions [8,9,10,11,32]. All these features are supported by DFT calculations employing a comparatively simple computational protocol (Table S1). Concerning the Ni ions, the complex of Ni(II) with two bidentate ligands is neutral, and the anticipated coordination is square planar or tetrahedral. The complex of Ni(IV) with the same ligands is neutralized with two Cl^−^ counterions, thus providing an octahedral coordination shell. Both complexes should be singlets in case the Ni(II) one is square planar. 

#### 2.1.1. Lithiation of Ni(II)(L1)_2_

**Figure 2:**
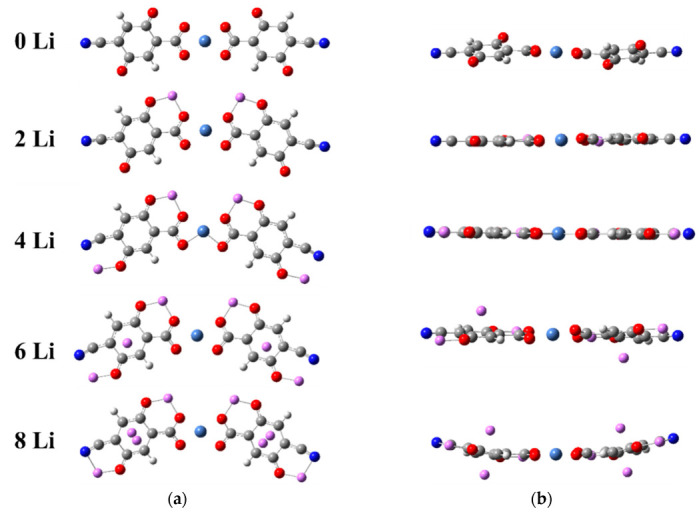
Stages of lithiation of Ni(II)(L1)_2_—top (**a**) and side (**b**) view of the optimized geometries. Color code: C—gray, H—white, O—red, N—deep blue, Ni—light blue, Li—pink.

The Ni(II)(L1)_2_ complex was optimized without symmetry restrictions, both as a singlet and a triplet. Both converged to a slightly twisted square planar geometry (Figure 2), the singlet being with lower energy (Table 1), so this is the basic structure to be reduced by Li further on.

**Figure 3:**
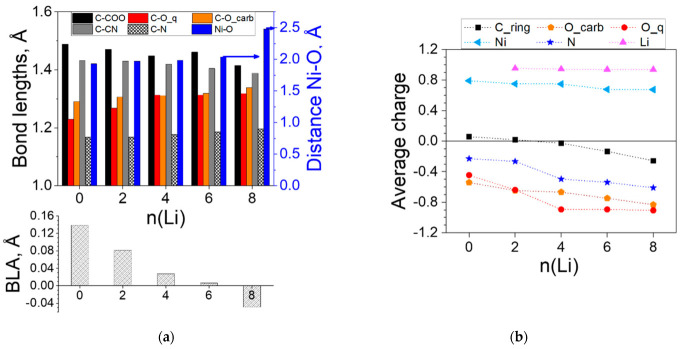
Ni(II)(L1)_2_ complex: (**a**) variation in the averaged bond lengths (Ni-O on the right axis) and bond length alternation (BLA); (**b**) averaged charges upon introduction of *n* Li atoms. Numerical values can be found in Tables S2 and S3.

Two Li atoms were added in the first step of reduction, testing three different initial geometries: both Li in one ligand and one Li in each ligand in two symmetries—C_2v_ and C_2h_. The first one was a singlet with higher energy than the other two. The latter two were triplets and converged to the same planar C_2v_ symmetry as shown in Figure 2. Each Li is coordinated by two oxygens—a quinone and a carboxyl one. The addition of the next two Li atoms resulted in the reduction of the second pair of quinone oxygens with partial involvement of the cyano-nitrogens. Two more Li atoms were located on the carbon rings, their C_2h_ configuration being preferred to the C_2v_ one, and the next two Li occupied the opposite position on the rings. The optimized geometry at this step (8Li) resulted in the loss of planarity, indicating that the maximum addition is reached. Indeed, further lithiation caused decoordination, so the complex was able to accommodate twice as many Li atoms compared to two free ligands. In the process of lithiation, the complex became perfectly planar and from linear turned slightly V-shaped, which resulted in the contraction of its length, measured as the distance between the two nitrogens (Table 1). The length alters due to the opposite effects of the emerging curvature (contraction) and decomplexation of Ni (extension), as shown in Figure 3a, but overall, the length varies insignificantly (3–4%).

Other structural changes occurring upon lithiation of the complex are the variation in the bond lengths within the ligands. The BLA characterizes the quinoid geometries, giving the difference between the average values of the long and the short C-C bonds in a quinone ring. As shown in Figure 3, the BLA is substantial in the empty complex, but quickly decreases with Li uptake due to aromatization of the rings and becomes negative at n = 8, meaning that the quinoidization changes its direction. Figure 3 also illustrates that the exocyclic C-C bonds gradually shorten while the C-N (slightly) and the C-O (sizably) bonds stretch, in support of the BLA data. The Ni-O distance slowly grows upon lithiation to 6Li and noticeably increases at 8Li indicating the beginning of the complex destruction. 

All these changes are reflected in the charge distribution (Figure 3b). The charge transfer from Li is almost complete—the average Natural Bond Orbital (NBO) charge of Li is constant at 0.95. The charge of Ni is also quite steady, decreasing from 0.8 to 0.7, disclosing essentially no participation of Ni(II) in the redox process. The electron density from Li is transferred most prominently to the oxygens, the carbonyl ones being more active in the first steps (0–4Li). The nitrogens come into play first at 4Li and so do the carbon rings.

#### 2.1.2. Lithiation of Ni(IV)(L1)_2_

The results obtained for the complex of Ni(II) demonstrate clearly that, unlike in the inorganic electrodes, here the transition metal takes no part in the redox process. In the complexes of Ni(IV), however, a different pattern was expected.

**Figure 4:**
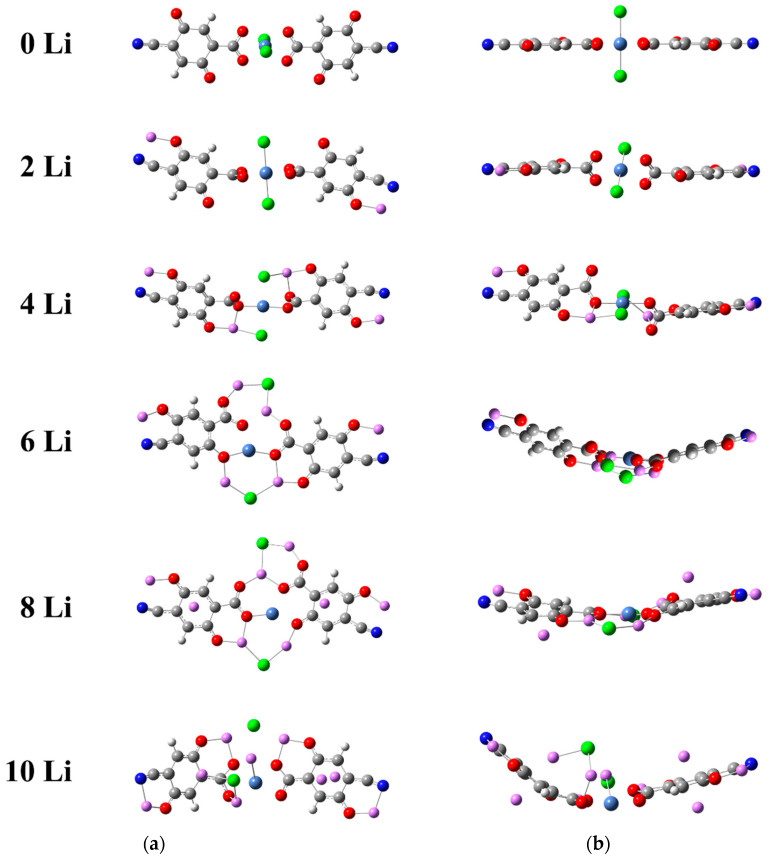
Stages of lithiation of Ni(IV)(L1)_2_—(**a**) top and (**b**) side view of the optimized geometries. Color code: C—gray, H—white, O—red, N—deep blue, Ni—light blue, Cl—green, Li—pink.

Same as for the Ni(II) complexes, at each stage of lithiation we tested different initial geometries and different multiplicities. Similarly, only the 2Li complex turned out triplet, just 6 kJ/mol apart from a singlet in which the two Li atoms are located close to the Cl^−^ ions (Figure S1). The obtained optimized geometries of the most stable models are presented in Figure 4, and the positions of the Li atoms are more or less the same as in Ni(II)(L1)_2_ for 2Li, 4Li, 8Li and 10Li. Only 6Li offers a new configuration due to the presence of Cl^−^. However, in no case was the release of LiCl from the complex established. 

**Figure 5:**
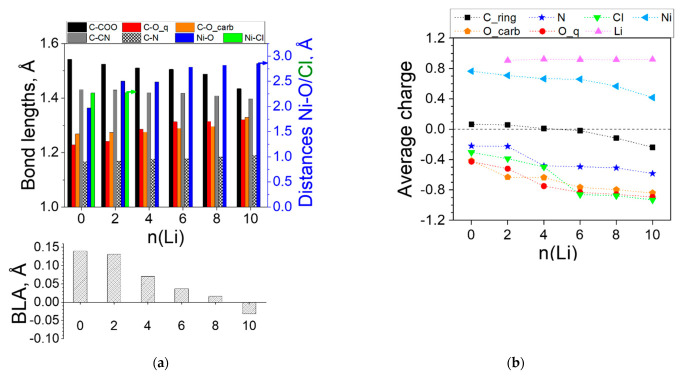
Ni(IV)(L1)_2_ complex: (**a**) variation in the averaged bond lengths (Ni-O and Ni-Cl on the right axis) and BLA; (**b**) averaged charges upon introduction of *n* Li atoms. Numerical values can be found in Tables S4 and S5. Distances Ni-Cl are not shown after 2Li in (**a**) as they are too large and not informative (Table S4).

It is visible that the empty complex is planar with an octahedral coordination of Ni(IV), Cl^−^ acting as a ligand, as justified by the distance to Ni and the NBO charges (Figure 5a,b). Upon lithiation, the planarity is disturbed (2Li, 4Li) and a prominent curvature emerges, reflected in the contraction of the N-to-N distance l_mol_—in this case, the contraction is more marked—about 10% (Table 2). The compensating effect of the increasing Ni-O distance observed in the Ni(II)(L1)_2_ complex does not apply to Ni(IV)(L1)_2_—although the average Ni-O values in Figure 5a appear quite large, two of the bonds persist about 1.9 Å at any degree of lithiation, while the other 2 (to the oxygens closer to the quinone ones) are longer than 3.5 Å, as these oxygens become part of the Li coordination shell. Analogous is the fate of the Cl^−^—as soon as Li is added, Cl^−^ turns from a ligand of nickel into a counterion of Li^+^, completely detached from Ni and with a growing negative charge (Figure 5a,b). Upon further addition of Li, decomplexation of Ni ensues. 

The remaining structural changes differ slightly from the Ni(II) case—a slower BLA decrease and a milder requinoidization only in the direction of the carboxyl group. The Li atoms become ions (charges > 0.9), transferring electron density predominantly to the oxygens of the ligands. The nitrogens and the ring carbons get involved first at 4Li. Unlike Ni(II), Ni(IV) also takes part in the charge redistribution, gradually reducing its positive charge, the profile being strictly parallel to that of the ring carbons.

#### 2.1.3. Redox Potentials of the Complexes Versus Li^+^/Li^0^

**Figure 6:**
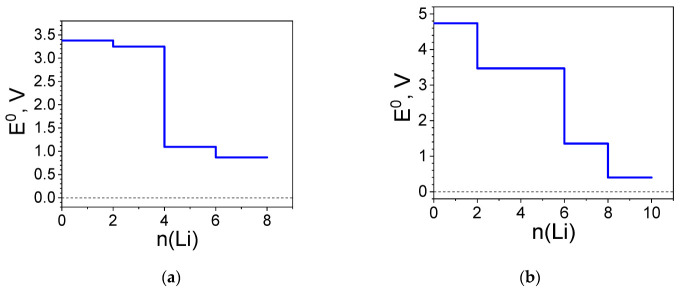
Redox potential profile (E^0^ vs. Li^+^/Li^0^) of: (**a**) Ni(II)(L1)_2_ and (**b**) Ni(IV)(L1)_2_ upon lithiation.

The calculated standard redox potential profiles for the two complexes are shown in Figure 6. For the Ni(II) complex, the redox potential profile clearly reproduces the structural changes and the redistribution of charges with a threshold number of Li (i.e., 4Li). The profile features two distinct plateaus—one of them at nearly 3.3 V and the other at 1.0 V. The first voltage plateau at 3.3 V resembles that calculated for the uncoordinated ligand (Table S1): 3.3 V versus 2.9 V, respectively. In addition, the reduction of Ni(II)(L1)_2_ with the first two pairs of Li takes place at very close potentials, this behavior being typical for para-BQ derivatives interacting with Li [49]. All these features signpost once again that Ni(II) does not take part in the redox reaction. The abrupt decrease in the redox potential from 3.3 V to 1.0 V is associated with an accommodation of extra Li, surpassing the uptake of the uncoordinated ligands. Instead of Ni(II), it is rather the quinoid ring carbons that take part in the further redox reaction (Figure 3b). 

The pattern of the gradual involvement of different components in the redox process in Ni(IV)(L1)_2_ is reflected in a different redox potential profile—instead of two distinct plateaus, the profile is of a staircase type, each degree of lithiation characterized by a different value of the potential (Figure 6b). This evidences the involvement of Ni(IV) in the redox reaction in addition to the BQ-based ligands. As a result, the initial potential reaches a value of 4.7 V, which is much higher than that of the isolated ligand. Moreover, this potential is commeasurable with the high-voltage inorganic electrode LiNi_1/2_Mn_3/2_O_4_ spinel (4.7 V) [7]. The Ni(IV) complex accommodates more Li atoms than the Ni(II) complex, reaching a low potential of 0.4 V. 

### 2.2. Coordination Polymers

As seen from the above results, the electrochemical parameters of the complexes are quite encouraging, but their structural flexibility is a major shortcoming. One way of circumventing this problem is to fix their structure by locking the complexes in a more rigid construction. Therefore, we considered modeling 1D coordination polymers of Ni(II) and Ni(IV) with quinone ligands in periodic boundary conditions.

#### 2.2.1. Coordination Polymers of Ni with 2,5-Dicarboxylato-1,4-benzoquinone (L2)

**Figure 7:**
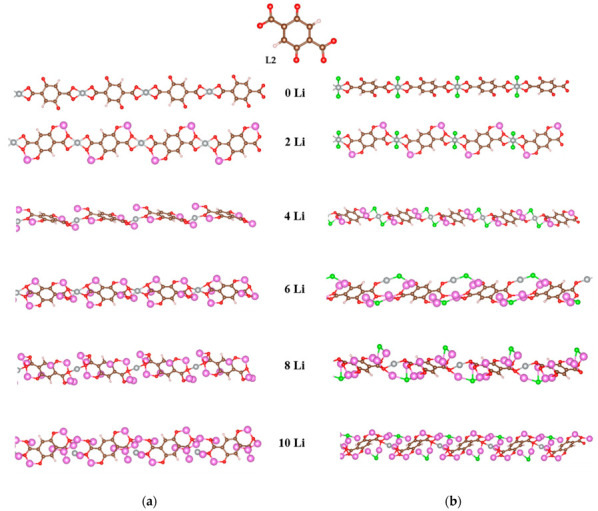
Optimized geometries of the step-by-step lithiated coordination polymers of (**a**) Ni(II) and (**b**) Ni(IV) with 2,5-dicarboxylato-1,4-quinone (L2). Color code: C—brown, H—white, O—red, Ni –gray, Cl—green, Li—pink.

The results obtained so far attest that the main participants in the redox reaction are the oxygen atoms in the carbonyl and carboxyl groups, so our first models contained only these functional groups; thus, ligand L2 is 2,5-dicarboxylato-1,4-benzoquinone (Figure 1b). The optimized structures of the coordination polymers of Ni(II) and Ni(IV) at the feasible degrees of lithiation are shown in Figure 7.

Indeed, in the first step of lithiation (2Li), only oxygens are involved in both oxidation states of nickel. The second pair of Li atoms (4Li) is coordinated at the same oxygens; this results in partial decoordination of Ni (in both states), which remains attached only to two oxygens. Although in the next step (6Li) the lithium atoms were placed initially on top of the carbon rings, they preferred again the proximity to oxygens in the Ni(II) polymer and Ni became three-coordinated by oxygen; in the Ni(IV) polymer, Li persisted at the rings but Ni remained two-coordinated by an oxygen and a Cl^−^. Further on (8Li), Ni coordination with two oxygens is restored and retained even at 10Li in the Ni(IV) structure, while completely lost in the Ni(II) one.

**Figure 8:**
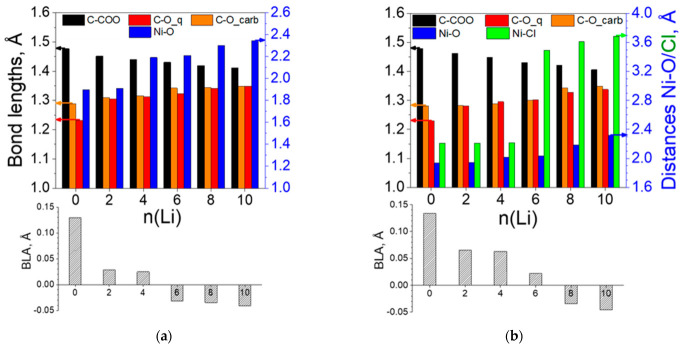
Variation in the averaged bond lengths and BLA of (**a**) [Ni(II)L2]_n_ and (**b**) [Ni(IV)L2]_n_. Arrows instruct which axis corresponds to the values of the structural parameters. Numerical quantities can be found in Tables S6 and S7.

**Figure 9:**
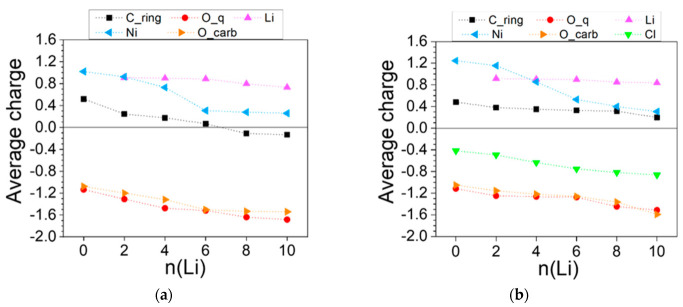
Averaged AIM charges in the lithiated (**a**) [Ni(II)L2]_n_ and (**b**) [Ni(IV)L2]_n_. Numerical values can be found in Tables S8 and S9.

As a result, the length of the elementary unit oscillates within 4% for both polymers (Table 3). Naturally, the length depends not only on the coordination of Ni but on the variation in all structural parameters. As seen in Figure 8, the BLA drops faster in [Ni(II)L2]_n_ due to a faster extension of the carbonyl bond. The growth of the C-O bonds in the carboxyl group, parallel to the shortening of the C-COO bond, indicates that after the aromatization of the ring, new quinoidization takes place, this time in direction of the carboxyl groups. Therefore, the BLA value becomes progressively negative. The Cl^−^ ions serve as ligands up to 4Li and are fully decoordinated from Ni thereafter, which is well supported by the charge values, which grow from −0.4 to −0.9 (Figure 9).

The charge profiles of the two polymers show both similarities and differences (Figure 9). The electron density transferred from Li is >0.9 in the first step (2Li) and slowly decreases upon further lithiation. At any degree of reduction, Li charge is higher in [Ni(IV)L2]_n_. The negative charge of oxygen progressively increases, steeper in the first steps for [Ni(II)L2]_n_ and the last steps for [Ni(IV)L2]_n_. Ni is being reduced more moderately in the first steps and with a leap at 6Li, in line with the observed decoordination. The carbon ring gradually accumulates electron density in [Ni(II)L2]_n_, switching from positive to negative values, which explains why when placed on top of the ring, Li remained there; in [Ni(IV)L2]_n_ it moved away, as the ring retained the overall positive charge—the released electron density from the metals was adopted by the chloride ligands instead. 

**Figure 10:**
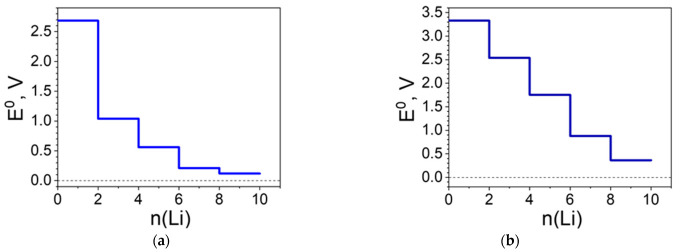
Redox potential profile (E^0^ vs. Li^+^/Li^0^) of: (**a**) [Ni(II)L2]_n_ and (**b**) [Ni(IV)L2]_n_ upon lithiation.

Both the bond length and charge profiles of [Ni(II)L2]_n_ reveal a more drastic change at 2Li and milder changes thereafter, so it is no surprise that after the initial value of 2.7 V, the potential profile features a radical drop of about 1.7 V, while from 2Li to 10Li the changes are less than 1V (Figure 10a). The monotonous variations in the structural data and charge distribution of [Ni(IV)L1]_n_ upon lithiation are reflected in a regular staircase profile of the potential with steps of ~0.8 V per two Li atoms added, the first step is at 3.3 V (Figure 10b). The initial potential of [Ni(IV)L2]_n_ is higher than that of [Ni(II)L2]_n_, as was observed for the respective complexes. It is of importance that in the initial and final stage of lithiation, the redox potentials for the coordination polymers are lower than that of the corresponding complexes. Taking into account the dissimilar computational approach and that the ligands are different in number and position of the functional groups, the direct comparison of complexes and polymers is illegitimate. Yet, when the ligand-to-metal ratio is 2:1, Ni(II) remains redox-innocent, while at a 1:1 ratio, Ni(II) is involved at a comparatively early lithiation stage (after 4Li).

#### 2.2.2. Coordination Polymers of Ni with 2,5-Dicarboxylato-3,6-dicyano-1,4-benzoquinone (L3)

**Figure 11:**
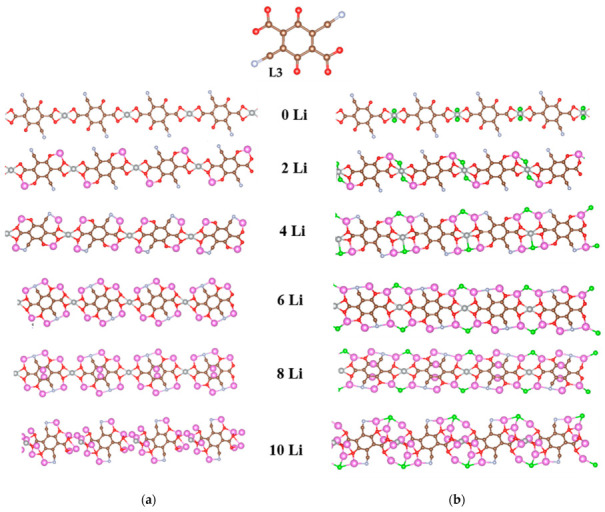
Optimized geometries of the step-by-step lithiated coordination polymers of (**a**) Ni(II) and (**b**) Ni(IV) with 2,5-dicarboxylato-3,6-dicyano-1,4-benzoquinone (L3). Color code: C—brown, H—white, O—red, Ni –gray-large, N—light blue-gray-small, Cl—green, Li—pink.

The results for the nickel coordination polymers with L2 are gratifying in respect of the impressive Li uptake (i.e., 10 Li atoms per elementary unit without destruction compared to 8-10 Li per two ligands in the isolated complexes) and the stable lower redox potentials. A further improvement would be the increase in the initial potential. As already seen, a viable strategy for increasing the potential is the introduction of more electron-withdrawing functional groups. Therefore, we modeled coordination polymers of Ni(II) and Ni(IV) with another ligand, containing two C≡N groups in the structure of L2, namely L3 (Figure 1c). The redox potential of L3 is higher than that of L2. The optimized geometries of these polymers upon stepwise lithiation are shown in Figure 11.

In [Ni(II)L3]_n_ the first two lithium atoms are bicoordinated by two oxygens each as in [Ni(II)L2]_n_, while in [Ni(IV)L3]_n_ Li is tricoordinated by two oxygens and a chloride ion even in this first step. At 4Li and 6Li the C≡N nitrogen is also involved in the Li coordination in both polymers, lithium being bicoordinated in [Ni(II)L2]_n_ and tricoordinated in [Ni(IV)L3]_n_. At 8Li the two newly added atoms were placed at the ring—above and below—and remained there while at 10Li, the coordination in both polymers cannot be strictly defined. Ni(II) is tetracoordinated at all stages of lithiation up to 8Li and essentially decoordinated at 10Li. In contrast, Ni(IV) stays tetracoordinated at all stages of lithiation considered. From 6Li on, however, Ni(IV) completely loses the chloride ligands, which are entirely ‘usurped’ by 2 or 3 lithiums.

Both polymers feature well-organized and compact structures due to the steady Ni coordination. This is reflected in the stable repeating unit length, which is remarkably constant in [Ni(IV)L3]_n_ and declines only at 10Li in [Ni(II)L3]_n_ (Table 4).

**Figure 12:**
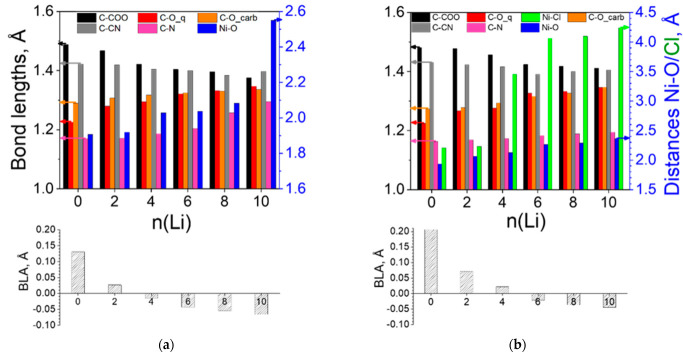
Variation in the averaged bond lengths and BLA of (**a**) [Ni(II)L3]_n_ and (**b**) [Ni(IV)L3]_n_. Arrows instruct which axis corresponds to the values of the structural parameters. Numerical quantities can be found in Tables S10 and S11.

The structural changes invoked by the Li uptake in [Ni(II)L3]_n_ (Figure 12a) resemble those in [Ni(II)L2]_n_ (Figure 8a), except for the more marked decrease in the C-COO bond and the less prominent shrinkage of the C-CN bond, while the C≡N and C-O bonds grow continuously. All these observations are in line with the BLA data indicating fast aromatization and further requinoidization along the C-COO and C-CN bonds, more expressed along the former. The presence of Cl^−^ ions in [Ni(IV)L3]_n_ slows down these effects versus [Ni(II)L3]_n_ but speeds them up compared to [Ni(IV)L2]_n_ (Figure 12b). The major structural changes occur during the first three steps (0-6Li) for both polymers, becoming milder upon further lithiation. 

**Figure 13:**
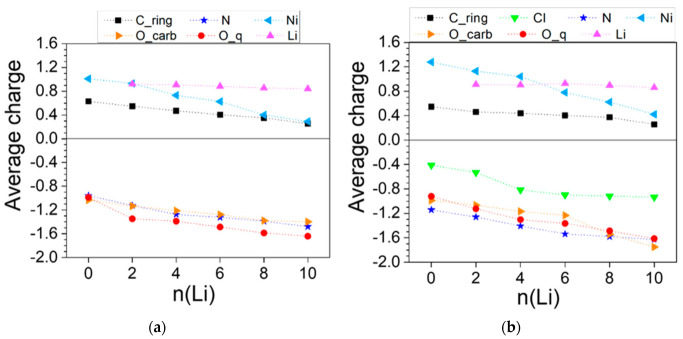
Averaged AIM charges in the lithiated (**a**) [Ni(II)L3]_n_ and (**b**) [Ni(IV)L3]_n_. Numerical values can be found in Tables S12 and S13.

The charge variation profiles of both polymers (Figure 13) reveal that while Li maintains a constant charge greater than 0.8 throughout the lithiation, the electron density at all other atoms grows in a fairly monotonous manner. In [Ni(II)L3]_n_ more distinct charge drops occur for the carbonyl oxygen at 2Li and for Ni at 4Li and 8Li (Figure 13a). In [Ni(IV)L3]_n_, charge drops are more discernable at 4Li for Cl and at 6-10Li for Ni (Figure 13b).

**Figure 14:**
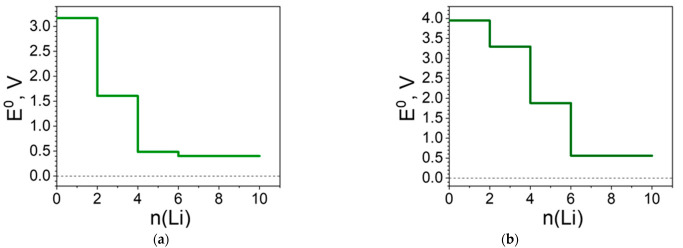
Redox potential profile (E^0^ vs. Li^+^/Li^0^) of: (**a**) [Ni(II)L3]_n_ and (**b**) [Ni(IV)L3]_n_ upon lithiation.

The structural and charge alterations are reflected in the redox potential profiles (Figure 14). As in all Ni(II) structures presented, the profile of [Ni(II)L3]_n_ is simpler with just three steps: the first (2Li) due predominantly to the carbonyl group reduction; the second, to the Ni involvement (Figure 14a). At this stage, the potential reaches the lowest value (i.e., at 0.4 V) and this potential remains nearly the same in a broad range of Li uptake (i.e., from 4 to 10 Li). The picture is different for [Ni(IV)L3]_n_ where the entire system is involved in the reduction. To the more prominent potential drops contribute mainly Cl at 4Li and Ni at 6Li (Figure 14b). Overall, the presence of the C≡N groups raises the initial potential by ca. 0.5 V (Figure 10 and Figure 14). 

### 2.3. Construction of an All-Organic Li-Ion Battery

Based on the calculated redox potentials and the Li uptake, the theoretical capacity and the gravimetric energy density are determined (Table 5). The Ni(IV)-bearing complex and polymers outperform the Ni(II) counterparts in terms of energy density. For all modeled polymers, as well as for the Ni(IV) complex, the lithiation was terminated at 10Li not because of potential drop to negative values but due to Ni decoordination symptoms. The decomplexation of Ni upon further addition of Li mimics the well-known conversion reaction occurring during Li-storing by transition metal oxides [50]. However, this process is beyond the scope of our study and, therefore, the number of Li atoms is restricted to 10, where the complex is still stable. It is worth noting that ten Li atoms per repeating unit in each polymer is an admirable number, signifying substantial capacity and energy density.

Another parameter of importance is the redox potential. The nickel complexes and polymers cover a broad range of redox potentials varying from the highest value at 4.7 V to the lowest one at 0.4 V. The large difference in the redox potentials is beneficial for the construction of an all-organic Li-ion battery from Ni complexes and Ni polymers. 

**Figure 15:**
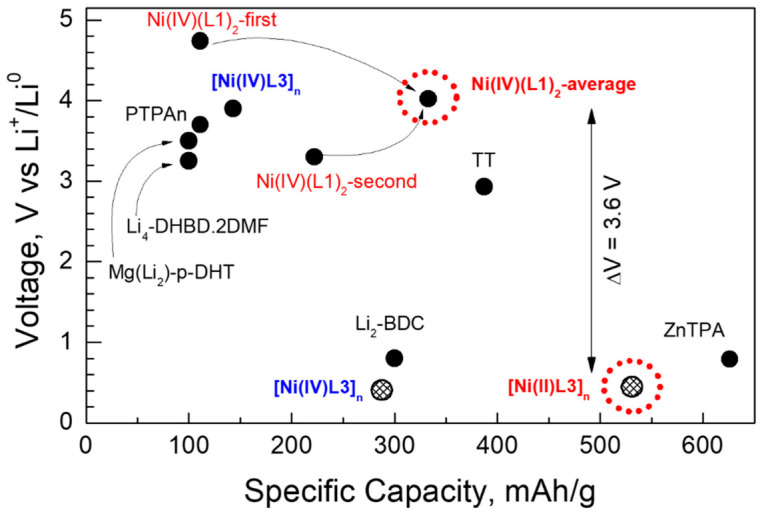
Voltage-capacity plot of organic-based electrode materials reported in the literature (PTPAn [51], Li_4_-p-DHBD.2DMF [47], Mg(Li_2_)-p-DHT [52], Li_2_-BDC [53] and ZnTPA [54]—black labels and circles), and in this study (colored labels, full circles for the high potential and patterned circles for the low potential); the most prospective structures designed in this study (in red) for the construction of an all-organic rechargeable Li-ion battery.

The comparison shows that the Ni(IV)-bearing organic compounds display higher redox potentials than their Ni(II) counterparts. This is a consequence of the more effective participation of Ni(IV) in the redox reaction with Li than Ni(II). Moreover, the Ni(IV)(L1)_2_ complex exhibits an initial redox potential of 4.7 V, which is among the highest potentials reported for organic compounds (Figure 15). The specific capacity delivered at this potential is also significant (111 mAh/g). It is noticeable that, at the second step, Ni(IV)(L1)_2_ also has promising properties: it delivers a much higher capacity (i.e., 222 mAh/g) at a redox potential which is still high (i.e., 3.3 V). Taking into account the first and second redox steps (Figure 6b), an average redox potential of 4.0 V and a specific capacity of 333 mAh/g are estimated. For the sake of comparison, one of the best organic cathodes, namely triptycene tribenzoquinone (TT) with a 3D structure, provides a specific capacity of 387 mAh/g at a redox potential of 2.9 V [49]. 

Contrary to the nickel complexes, the nickel polymers permit lower redox potentials to be reached at the final stage of lithiation. Among all nickel polymers and complexes, [Ni(II)L3]_n_ polymer displays the best properties: the redox potential is 0.45 V and the specific capacity is 531 mAh/g (Figure 15). These parameters are close to the best properties reported in the literature for ZnTPA: 0.79 V and 626 mAh/g [55]. 

Based on the highest and lowest redox potentials of the designed metal-organic constructs, an all-organic Li-ion battery could be assembled from Ni(IV)(L1)_2_ as a cathode and [Ni(II)L3]_n_ as an anode. This battery would operate at an average voltage exceeding 3.0 V by delivering a capacity of more than 300 mAh/g. For the sake of comparison, one of the best all-organic batteries consisting of two redox polymers, poly(2-vinylthianthrene) and poly(2-methacrylamide-TCAQ), is characterized by an operating voltage higher than 1.3 V and a theoretical capacity of more than 100 mAh/g [55]. 

## 3. Methods

All calculations were performed using the density functional theory. The calculations of the complexes in the gas phase were carried out with the Gaussian 16 [56] program, using the Becke approximation for the exchange energy [57] and the Lee–Yang–Parr approximation for the correlation energy [58] with a triple-zeta Pople-style basis set 6-311++G** [59,60,61,62]. This protocol was chosen as it provides good reproducibility of the experimental values for the electrode potential of quinones (Table S14). The optimized geometries were true minima on the potential energy surface confirmed by frequency analysis. The thermochemistry was estimated at 298.15 K. The charge transfer was assessed by NBO analysis [63,64]. 

The calculations of the 1D-coordination polymers were carried out in periodic boundary conditions using the VASP 5.4.4 program [65,66,67] with the spin-polarized PBE generalized gradient approximation [68] and the projector-augmented wave (PAW) method [69,70]. A plane-wave energy cut-off of 700 eV was applied and the Γ-point was used to sample the Brillouin zone. The electronic partial occupancies were calculated according to the Gaussian smearing scheme with a smearing parameter of 0.05 eV. This protocol was found functional in our previous studies [43]. The positions of the atoms were relaxed until all forces acting on the atoms became less than 0.01 eV/Å. The charge distribution in the periodic calculation was quantified with the Bader program [71] according to Bader’s quantum theory of atoms in molecules (QTAIM) [72]. The VESTA program [73] was used for structure visualization.

The electrochemical potential at each step of lithiation was calculated according to the equations:

ΔG_DFT_ = G(Li_y_MOC) − [G(Li_x_MOC) + (y − x)G(Li)] (1)

E^0^ = −ΔG_DFT_ /zF (2)

where x and y are the number of lithium atoms in two consecutive steps of metal insertion, z is the number of electrons transferred, F is the Faraday constant, G is the equilibrium free energy, and G(Li) is the energy of one Li atom in the gas phase. The utilization of Li_(g)_ instead of Li_(s)_ as a reference is justified by benchmark calculations (see Table S14) which show very large deviations with respect to the experiment when the extrapolated energy of one lithium atom in the solid state is used. Therefore, G(Li_(g)_) was used as a reference value. Concerning the calculation of the coordination polymers, it has been shown that for solid systems, the difference between the internal and the free energy is negligibly small and the electrode potential can be estimated using the former instead of the latter [74,75], which was utilized for the periodic structures. 

The capacity and the energy density of the electrode material were calculated according to the expressions:

Capacity = zF/(3.6M)(3)

Energy density = F**∫**Edz/(3.6M) (4)

with M being the molar mass of the material.

## 4. Conclusions

The purpose of this study was the design of hybrid electrode materials for lithium-ion batteries combining transition metal ions with organic redox-active counterions. Para-BQs, traditionally undergoing reduction by two Li atoms to convert into aromatic lithium derivatives of benzohydroquinone, were functionalized with electron-withdrawing groups and used as ligands in complexes and coordination polymers of Ni in two oxidation states: Ni(II) and Ni(IV). Molecular modeling with first principles quantum-mechanical methods was utilized: BLYP/6-311++G** for the complexes and PBE/PAW in periodic boundary conditions for the polymers. The obtained structures exhibited a propensity to interact with a markedly higher number of Li atoms compared to their building moieties alone.

The analysis of the obtained results revealed features that can be summarized as follows: 

In the complexes, the C=O-containing functional groups are the most efficient electron-acceptors, the C≡N becoming active at a later stage of Li insertion and Ni coming into play last (if at all). The Cl^−^ ligands in the complex of Ni(IV) adopt substantial amounts of electron density, which slows down the aromatization of the quinones and allows a higher number of Li atoms to be accommodated. The incorporation of redox-active ligands into complexes with transition metal ions increases the Li uptake per ligand and favorably enhances the electrochemical characteristics but invokes unwanted structural deformations and decomplexation of Ni.

Locking the geometry by constructing 1D coordination polymers improves the structural stability for both oxidation states of Ni—the polymers retain planarity and demonstrate negligible variation in the unit cell length. When present, the C≡N nitrogen is as active as the oxygens with respect to electron density redistribution. Although the reduction in all systems under study is initiated in the organic fragment, in the polymers, nickel also starts being involved at an early stage.

The electrochemical potential profiles of all Ni(II) structures are simple, with one or two distinct drops. Those of Ni(IV) are more complex, with specific (almost equidistant) values for each degree of lithiation. The higher redox potentials are achieved for the Ni(IV)-bearing organic compounds due to their more effective participation in the reaction with Li. Among all designed complexes and polymers, Ni(IV)(L1)_2_ delivers a relatively good capacity at a high potential, while [Ni(II)L3]_n_ is characterized by a stable low potential and large capacity. Thus, Ni(IV)(L1)_2_ and [Ni(II)L3]_n_ could serve as a cathode and an anode, respectively, in an all-organic Li-ion battery.

## Figures and Tables

**Table 1:** Multiplicity and N-to-N length (l_mol_) of Ni(II)(L1)_2_ upon lithiation.

n(Li)	0	2	4	6	8
multiplicity	S	T	S	S	S
l _mol_, Å	18.6	18.5	18.2	18.1	18.0

**Table 2:** Multiplicity and N-to-N length (l_mol_) of Ni(IV)(L1)_2_ upon lithiation.

n(Li)	0	2	4	6	8	10
multiplicity	S	T	S	S	S	S
l_mol_, Å	19.0	19.0	18.8	18.0	17.3	17.0

**Table 3:** Elementary unit length (l_eu_) of the polymers with L2 upon lithiation.

n(Li)	0	2	4	6	8	10
[Ni(II)L2]_n_ l_eu_, Å	8.86	8.87	8.91	8.56	8.56	8.73
[Ni(II)L2]_n_ l_eu_, Å	8.91	8.83	8.59	8.69	8.70	8.70

**Table 4:** Repeating unit length (l_eu_) of the polymers with L3 upon lithiation.

n(Li)	0	2	4	6	8	10
[Ni(II)L3]_n_ l_eu_, Å	8.98	8.97	8.93	8.95	8.87	8.24
[Ni(IV)L3]_n_ l_eu_, Å	8.93	8.92	8. 96	8.94	8.95	8.97

**Table 5:** Summarized electrochemical parameters of the designed constructs.

Coordination Compounds	ΔE^0^ (Initial), V	n_max_	Capacity, mA.h.g^−1^	Energy Density, W.h.g^−1^
Ni(II)(L1)_2_	3.38	8	522	1121
Ni(IV)(L1)_2_	4.74	10	556	1495
[Ni(II)L2]_n_	2.68	10	1 061	977
[Ni(IV)L2]_n_	3.33	10	828	1467
[Ni(II)L3]_n_	3.17	10	885	1073
[Ni(IV)L3]_n_	3.95	10	717	1469

## Data Availability

The input and output files from the calculations (including geometries and energies) are available from the corresponding authors upon reasonable request.

## Supplementary Materials

The following are available online at https://www.mdpi.com/article/10.3390/molecules27206805/s1, Figure S1: Alternative structure of the Ni(IV)(L1)_2__2Li complex; Table S1: Calculated electrochemical potential of quinone-based redox-active species; Table S2: BLA and averaged bond lengths in the lithiated Ni(II)(L1)_2_; Table S3: Averaged NBO charges in the lithiated Ni(II)(L1)_2_; Table S4: BLA and averaged bond lengths in the lithiated Ni(IV)(L1)_2_; Table S5: Averaged NBO charges in the lithiated Ni(IV)(L1)_2_; Table S6: BLA and averaged bond lengths in the lithiated [Ni(II)L2]_n_; Table S7: BLA and averaged bond lengths in the lithiated [Ni(IV)L2]_n_; Table S8: Averaged AIM charges in the lithiated [Ni(II)L2]_n_; Table S9: Averaged AIM charges in the lithiated [Ni(IV)L2]_n_; Table S10: BLA and averaged bond lengths in the lithiated [Ni(II)L3]_n_; Table S11: BLA and averaged bond lengths in the lithiated [Ni(IV)L3]_n_; Table S12: Averaged AIM charges in the lithiated [Ni(II)L3]_n_; Table S13: Averaged AIM charges in the lithiated [Ni(IV)L3]_n_; Table S14: Comparison between experimental and calculated electrochemical potentials of quinone-based molecules. Refs. [49,76,77,78] are cited in the Supplementary Materials.

## References

[1] Larcher D., Tarascon J.M. (2015). Towards greener and more sustainable batteries for electrical energy storage. Nat. Chem..

[2] Poizot P., Gaubicher J., Renault S., Dubois L., Liang Y., Yao Y. (2020). Opportunities and Challenges for Organic Electrodes in Electrochemical Energy Storage. Chem. Rev..

[3] Schon T.B., McAllister B.T., Li P.-F., Seferos D.S. (2016). The rise of organic electrode materials for energy storage. Chem. Soc. Rev..

[4] Lu Y., Zhang Q., Li L., Niu Z., Chen J. (2018). Design Strategies toward Enhancing the Performance of Organic Electrode Materials in Metal-Ion Batteries. Chemistry.

[5] Lu Y., Chen J. (2020). Prospects of organic electrode materials for practical lithium batteries. Nat. Rev. Chem..

[6] Esser B., Dolhem F., Becuwe M., Poizot P., Vlad A., Brandell D. (2021). A perspective on organic electrode materials and technologies for next generation batteries. J. Power Sources.

[7] Whittingham M.S. (2004). Lithium Batteries and Cathode Materials. Chem. Rev..

[8] Li Q., Zhou D., Zhang L., Ning D., Chen Z., Xu Z., Gao R., Liu X., Xie D., Schumacher G. (2019). Tuning Anionic Redox Activity and Reversibility for a High-Capacity Li-Rich Mn-Based Oxide Cathode via an Integrated Strategy. Adv. Funct. Mater..

[9] Gottis S., Barrès A.-L., Dolhem F., Poizot P. (2014). Voltage Gain in Lithiated Enolate-Based Organic Cathode Materials by Isomeric Effect. ACS. Appl. Mater. Interfaces.

[10] Friebe C., Lex-Balducci A., Schubert U.S. (2019). Sustainable Energy Storage: Recent Trends and Developments toward Fully Organic Batteries. ChemSusChem.

[11] Lakraychi A.E., Dolhem F., Vlad A., Becuwe M. (2021). Organic Negative Electrode Materials for Metal-Ion and Molecular-Ion Batteries: Progress and Challenges from a Molecular Engineering Perspective. Adv. Energy Mater..

[12] Wang D.-Y., Liu R., Guo W., Li G., Fu Y. (2021). Recent advances of organometallic complexes for rechargeable batteries. Coord. Chem. Rev..

[13] Hogue R.W., Toghill K.E. (2019). Metal Coordination Complexes in Non-Aqueous Redox Flow Batteries. Curr. Opin. Electrochem..

[14] Hirao T. (2002). Conjugated systems composed of transition metals and redox-active π-conjugated ligands. Coord. Chem. Rev..

[15] Kaim W., Schwederski B. (2010). Non-innocent ligands in bioinorganic chemistry—An overview. Coord. Chem. Rev..

[16] Broere D.L.J., Plessius R., der Vlugt J.I. (2015). New avenues for ligand-mediated processes–expanding metal reactivity by the use of redox-active catechol, o-aminophenol and o-phenylenediamine ligands. Chem. Soc. Rev..

[17] Pierpont C.G., Buchanan R.M. (1981). Transition metal complexes of o-benzoquinone, o-semiquinone, and catecholate ligands. Coord. Chem. Rev..

[18] Kaim W. (1987). The Transition Metal Coordination Chemistry of anion radicals. Coord. Chem. Rev..

[19] Pierpont C.G. (2001). Unique properties of transition metal quinone complexes of the MQ_3_ series. Coord. Chem. Rev..

[20] Poddel’sky A.I., Cherkasov V.K., Abakumov G.A. (2009). Transition metal complexes with bulky 4,6-di-*tert*-butyl-N-aryl(alkyl)-o-iminobenzoquinonato ligands: Structure, EPR and magnetism. Coord. Chem. Rev..

[21] Kaim W. (2011). Manifestations of Noninnocent Ligand Behavior. Inorg. Chem..

[22] Tezgerevska T., Alley K.G., Boskovic C. (2014). Valence tautomerism in metal complexes: Stimulated and reversible intramolecular electron transfer between metal centers and organic ligands. Coord. Chem. Rev..

[23] Kaim W., Paretzki A. (2017). Interacting metal and ligand based open shell systems: Challenges for experiment and theory. Coord. Chem. Rev..

[24] Pashanova K.I., Poddel’sky A.I., Piskunov A.V. (2022). Complexes of “late” transition metals of the 3d row based on functionalized o-iminobenzoquinone type ligands: Interrelation of molecular and electronic structure, magnetic behaviour. Coord. Chem. Rev..

[25] Liu Q., Sleightholme A., Shinkle A., Li Y., Thompson L. (2009). Non-aqueous vanadium acetylacetonate electrolyte for redox flow batteries. Electrochem. Commun..

[26] Liu Q., Shinkle A., Li Y., Monroe C., Thompson L., Sleightholme A. (2010). Nonaqueous chromium acetylacetonate electrolyte for redox flow batteries. Electrochem. Commun..

[27] Mun J., Lee M.-J., Park J., Oh D.-J., Lee D.-Y., Doo S.-G. (2012). Non-Aqueous Redox Flow Batteries with Nickel and Iron Tris(2,2′-bipyridine) Complex Electrolyte. Electrochem. Solid-State Lett..

[28] Armstrong C., Toghill K. (2017). Cobalt(II) complexes with azole-pyridine type ligands for non-aqueous redox-flow batteries: Tunable electrochemistry via structural modification. J. Power Sources.

[29] Kim J.-H., Kim K.J., Park M.-S., Lee N.J., Hwang U., Kim H., Kim Y.-J. (2011). Development of metal-based electrodes for non-aqueous redox flow batteries. Electrochem. Commun..

[30] Burnea F., Shi H., Ko K., Lee J. (2017). Reduction potential tuning of first row transition metal M^III^/M^II^ (M = Cr, Mn, Fe, Co, Ni) hexadentate complexes for viable aqueous redox flow battery catholytes: A DFT study. Electrochim. Acta.

[31] Hwang S., Kim H., Ryu J.H., Oh S.M. (2017). Ni(II)-chelated thio-crown complex as a single redox couple for non-aqueous flow batteries. Electrochem. Commun..

[32] Alt H., Binder H., Kőhling A., Sandstede G. (1972). Investigation into the use of quinone compounds-for battery cathodes. Electrochim. Acta.

[33] Häupler B., Wild A., Schubert U. (2015). Carbonyls: Powerful Organic Materials for Secondary Batteries. Adv. Energy Mater..

[34] Araujo R.B., Banerjee A., Panigrahi P., Yang L., Strømme M., Sjödin M., Araujo C.M., Ahuja R. (2017). Designing strategies to tune reduction potential of organic molecules for sustainable high capacity battery application. J. Mater. Chem. A.

[35] Lee S., Hong J., Kang K. (2020). Redox-Active Organic Compounds for Future Sustainable Energy Storage System. Adv. Energy Mater..

[36] Lin Z., Shi H., Lin L., Yang X., Wu W., Sun X. (2021). A high capacity small molecule quinone cathode for rechargeable aqueous zinc-organic batteries. Nat. Commun..

[37] Miao L., Liu L., Shang Z., Li Y., Lu Y., Cheng F., Chen J. (2018). The structure–electrochemical property relationship of quinone electrodes for lithium-ion batteries. Phys. Chem. Chem. Phys..

[38] Wu Y., Zeng R., Nan J., Shu D., Qiu Y., Chou S.-L. (2017). Quinone Electrode Materials for Rechargeable Lithium/Sodium Ion Batteries. Adv. Energy Mater..

[39] Emanuelsson R., Sterby M., Strømme M., Sjödin M. (2017). An All-Organic Proton Battery. J. Am. Chem. Soc..

[40] Oka K., Strietzel C., Emanuelsson R., Nishide H., Oyaizu K., Strømme M., Sjödin M. (2019). Characterization of PE-DOT-Quinone conducting redox polymers in water-in-salt electrolytes for safe and high-energy Li-ion batteries. Electrochem. Commun..

[41] Ding B., Solomon M., Leong C., D’Alessandro D. (2021). Redox-active ligands: Recent advances towards their incorporation into coordination polymers and metal-organic frameworks. Coord. Chem. Rev..

[42] Baumann A.E., Burns D.A., Liu B., Thoi V.S. (2019). Metal-organic framework functionalization and design strategies for advanced electrochemical energy storage devices. Commun. Chem..

[43] Rasheev H., Seremak A., Stoyanova R., Tadjer A. (2022). Redox Hyperactive MOF for Li^+^, Na^+^ and Mg^2+^ Storage. Molecules.

[44] Manecke G., Storck W. (1963). Polyvinylanthraquinone redox resins (electron exchange polymers). J. Polym. Sci. Part C Polym. Symp..

[45] Xiang J., Chang C., Li M., Wu S., Yuan L., Sun J. (2008). A Novel Coordination Polymer as Positive Electrode Material for Lithium Ion Battery. Cryst. Growth Des..

[46] Chang C.-H., Li A.-C., Popovs I., Kaveevivitchai W., Chen J.-L., Chou K.-C., Kuo T.-S., Chen T.-H. (2019). Elucidating Metal and Ligand Redox Activities of Copper-Benzoquinoid Coordination Polymer as Cathode for Lithium-Ion Batteries. J. Mater. Chem. A.

[47] Lakraychi A.E., Deunf E., Fahsi K., Jimenez P., Bonnet J.-P., Djedaini-Pilard F., Bécuwe M., Poizot P., Dolhem F. (2018). An air-stable lithiated cathode material based on a 1,4-benzenedisulfonate backbone for organic Li-ion batteries. J. Mater. Chem. A.

[48] Xie J., Lu Y. (2021). Towards practical organic batteries. Nat. Mater..

[49] Kwon J.E., Hyun C.-S., Ryu Y.J., Lee J., Min D.J., Park M.J., An B.-K., Park S.Y. (2018). Triptycene-Based Quinone Molecules Showing Multi-Electron Redox Reactions for Large Capacity and High Energy Organic Cathode Materials in Li-Ion Batteries. J. Mater. Chem. A.

[50] Cao K., Jing T., Yang L., Jiao L. (2017). Recent progress in conversion reaction metal oxide anodes for Li-ion batteries. Mater. Chem. Front..

[51] Qin J., Lan Q., Liu N., Men F., Wang X., Song Z., Zhan H. (2019). A Metal-Free Battery with Pure Ionic Liquid Electrolyte. iScience.

[52] Jouhara A., Dupré N., Gaillot A.-C., Guyomard D., Dolhem F., Poizot P. (2018). Raising the redox potential in carboxyphenolate-based positive organic materials via cation substitution. Nat. Commun..

[53] Armand M., Grugeon S., Vezin H., Laruelle S., Ribiere P., Poizot P., Tarascon J.M. (2009). Conjugated dicarboxylate anodes for Li-ion batteries. Nat. Mater..

[54] Wild A., Strumpf M., Häupler B., Hager M., Schubert U. (2016). All-Organic Battery Composed of Thianthrene- and TCAQ-Based Polymers. Adv. Energy Mater..

[55] Wang L., Zou J., Chen S., Yang J., Qing F., Gao P., Li J. (2017). Zinc Terephthalates ZnC_8_H_4_O_4_ as Anodes for Lithium Ion Batteries. Electrochim. Acta.

[56] Frisch M.J., Trucks G.W., Schlegel H.B., Scuseria G.E., Robb M.A., Cheeseman J.R., Scalmani G., Barone V., Petersson G.A., Nakatsuji H. (2016). Gaussian 16, Revision, B.01.

[57] Becke A. (1988). Density-functional exchange-energy approximation with correct asymptotic behavior. Phys. Rev. A.

[58] Lee C., Yang W., Parr R. (1988). Development of the Colle-Salvetti correlation-energy formula into a functional of the electron density. Phys. Rev. B.

[59] McLean A.D., Chandler G.S. (1980). Contracted Gaussian-basis sets for molecular calculations. 1. 2^nd^ row atoms, Z = 11–18. J. Chem. Phys..

[60] Raghavachari K., Binkley J.S., Seeger R., Pople J.A. (1980). Self-Consistent Molecular Orbital Methods. 20. Basis set for correlated wave-functions. J. Chem. Phys..

[61] Wachters A.J.H. (1970). Gaussian basis set for molecular wavefunctions containing third-row atoms. J. Chem. Phys..

[62] Hay P.J. (1977). Gaussian basis sets for molecular calculations–representation of 3D orbitals in transition-metal atoms. J. Chem. Phys..

[63] Weinhold F., Foster J. (1980). Natural hybrid orbitals. J. Am. Chem. Soc..

[64] Glendening E., Reed A., Carpenter J., Weinhold F. (2003). NBO.

[65] Kresse G., Hafner J. (1993). Ab Initio Molecular Dynamics for Liquid Metals. Phys. Rev. B Condens. Matter Mater. Phys..

[66] Kresse G., Furthmüller J. (1996). Efficient Iterative Schemes for Ab Initio Total-Energy Calculations Using a Plane-Wave Basis Set. Phys. Rev. B Condens. Matter Mater. Phys..

[67] Kresse G., Furthmüller J. (1996). Efficiency of Ab-initio Total Energy Calculations for Metals and Semiconductors Using a Plane-Wave Basis Set. Comput. Mat. Sci..

[68] Perdew J.P., Burke K., Ernzerhof M. (1996). Generalized Gradient Approximation Made Simple. Phys. Rev. Lett..

[69] Blochl P.E. (1994). Projector augmented-wave method. Phys. Rev. B.

[70] Kresse G., Joubert D. (1999). From Ultrasoft Pseudopotentials to the Projector Augmented-Wave Method. Phys. Rev. B.

[71] Tang W., Sanville E., Henkelman G. (2009). A Grid-Based Bader Analysis Algorithm Without Lattice Bias. J. Phys. Condens. Matter.

[72] Bader R.F.W. (1985). Atoms in Molecules. Acc. Chem. Res..

[73] Momma K., Izumi F. (2011). VESTA 3 for three-dimensional visualization of crystal, volumetric and morphology data. J. Appl. Crystallogr..

[74] Aydinol M.K., Kohan A.F., Ceder G., Cho K., Joannopoulos J. (1997). Ab Initio Study of Lithium Intercalation in Metal Oxides and Metal Dichalcogenides. Phys. Rev. B Condens. Matter Mater. Phys..

[75] Araujo R.B., Banerjee A., Ahuja R. (2017). Divulging the Hidden Capacity and Sodiation Kinetics of Na_x_C_6_Cl_4_O_2_: A High Voltage Organic Cathode for Sodium Rechargeable Batteries. J. Phys. Chem. C.

[76] Xu D., Liang M., Qi S., Sun W., Lv L.-P., Du F.-H., Wang B., Chen S., Wang Y., Yu Y. (2021). The Progress and Prospect of Tunable Organic Molecules for Organic Lithium-Ion Batteries. ACS Nano.

[77] Lee J., Kim H., Park M.J. (2016). Long-life, high-rate lithium-organic batteries based on naphthoquinone derivatives. Chem. Mater..

[78] Yao Z., Tang W., Wang X., Wang C., Yang C., Fan C. (2020). Synthesis of 1,4-benzoquinone dimer as a high-capacity (501 mAhg−1) and high-energy-density (>1000 Whkg−1) organic cathode for organic Li-Ion full batteries. J. Power Sources.

