# Supplementary material for: Molecular Engineering of Quinone-Based Nickel Complexes and Polymers for All-Organic Li-Ion Batteries
Source: Molecules. 2022 Oct 11;27(20):6805. doi: 10.3390/molecules27206805 (PMC9608464; doi:10.3390/molecules27206805)
Supplement: Supplementary file 1 [file molecules-27-06805-s001.zip › molecules-1945171-supplementary.pdf]

Supplementary Materials for:

Molecular Engineering of Quinone-based Nickel Complexes and Polymers for All-Organic Li-ion Batteries

Yanislav Danchovski,[1,2] Hristo Rasheev,[1,2] Radostina Stoyanova[2,*] and Alia Tadjer[1,2,*]

[1] Faculty of Chemistry and Pharmacy, University of Sofia, 1164 Sofia, Bulgaria

[2] Institute of General and Inorganic Chemistry, Bulgarian Academy of Sciences, Sofia 1113, Bulgaria

e-mail: *tadjer@chem.uni-sofia.bg, radstoy@svr.igic-bas.bg*

Table S1: Calculated electrochemical potentials (at the BLYP/6-311++G** level of theory) vs. Li^+/Li^0 in solid and gas phase of some quinone-based redox-active species.

Molecule	ΔG^0_{sol}, kJ/mol	$\Delta E^0_{sol}, V$	ΔG^0_{gas}, kJ/mol	$\Delta E^0_{gas}, V$
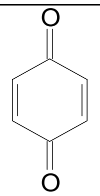	-183.10	0.949	-436.30	2.261
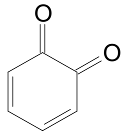	-210.26	1.090	-463.46	2.402
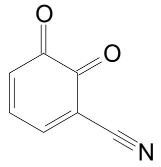	-403.64	2.092	-656.84	3.404
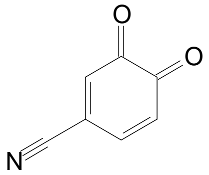	-266.25	1.380	-519.45	2.692
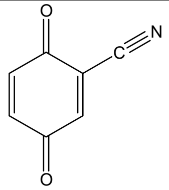	-296.16	1.535	-549.36	2.847

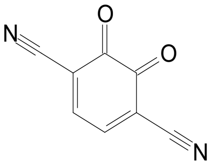	-420.95	2.181	-674.15	3.494
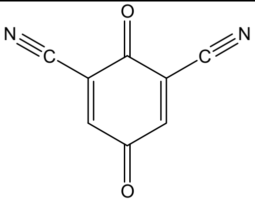	-401.27	2.079	-654.47	3.392
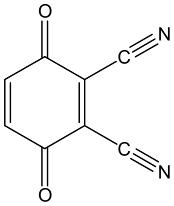	-401.26	2.079	-654.46	3.391
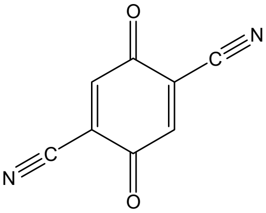	-387.44	2.008	-640.64	3.320
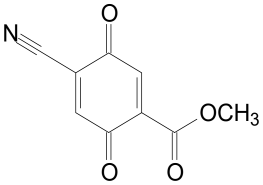	-405.59	2.102	-658.79	3.414
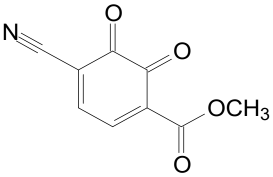	-445.92	2.311	-699.12	3.623
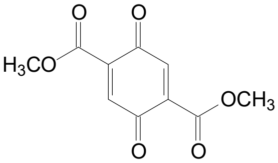	-425.49	2.205	-678.69	3.517
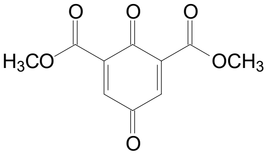	-336.63	1.744	-589.83	3.057

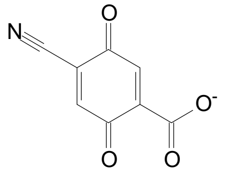 L1	-312.77	1.621	-565.96	2.933
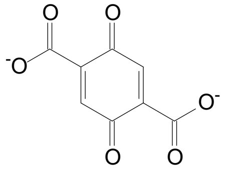 L2	-314.56	1.630	-567.75	2.942
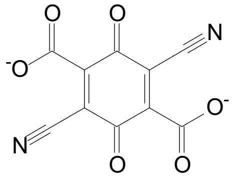 L3	-321.39	1.665	-574.58	2.978

Table S2: BLA and averaged bond lengths in the lithiated Ni(II)$(L1)_2$

n(Li)	BLA_av	C-COO	C-CN	CO_carb	CO_quin	C-N	Ni-O
0	0.14	1.49	1.43	1.29	1.23	1.17	1.93
2	0.08	1.47	1.43	1.31	1.27	1.17	1.97
4	0.03	1.45	1.42	1.31	1.31	1.18	1.99
6	0.01	1.46	1.41	1.32	1.31	1.19	2.04
8	-0.05	1.42	1.39	1.34	1.32	1.20	2.48

Table S3: Averaged NBO charges in the lithiated Ni(II)$(L1)_2$

n(Li)	C_ring	O_carbox	O_quin	N	Ni	Li
0	0.059	-0.542	-0.445	-0.230	0.790	
2	0.017	-0.648	-0.639	-0.265	0.750	0.953
4	-0.028	-0.668	-0.895	-0.497	0.747	0.942
6	-0.135	-0.749	-0.894	-0.540	0.676	0.938
8	-0.258	-0.833	-0.908	-0.611	0.674	0.937

Table S4: BLA and averaged bond lengths in the lithiated Ni(IV)$(L1)_2$

n(Li)	BLA_av	C-COO	C-CN	CO_carb	CO_quin	C-N	Ni-O	Ni-Cl
0	0.14	1.55	1.43	1.27	1.23	1.17	1.97	2.27
2	0.13	1.52	1.43	1.27	1.24	1.17	2.50	2.27
4	0.07	1.52	1.42	1.28	1.29	1.18	2.49	6.25
6	0.04	1.50	1.42	1.29	1.31	1.18	2.78	6.23
8	0.02	1.49	1.41	1.29	1.31	1.18	2.82	6.22
10	-0.03	1.42	1.39	1.34	1.31	1.19	2.85	6.29

Table S5: Averaged NBO charges in the lithiated Ni(IV)(L1)$_2$

n(Li)	C_ring	O_carbox	O_quin	N	Ni	Cl	Li
0	0.065	-0.417	-0.426	-0.220	0.764	-0.305	
2	0.057	-0.632	-0.520	-0.226	0.709	-0.388	0.907
4	0.009	-0.636	-0.749	-0.482	0.664	-0.497	0.923
6	-0.019	-0.766	-0.832	-0.494	0.659	-0.861	0.918
8	-0.117	-0.797	-0.859	-0.509	0.566	-0.876	0.917
10	-0.239	-0.840	-0.892	-0.583	0.417	-0.931	0.919

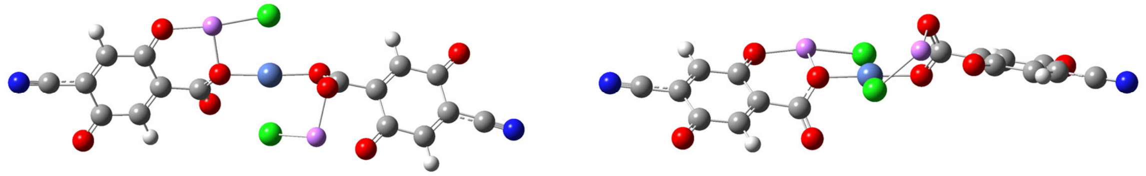

Fig. S1: Alternative structure of the Ni(IV)_2Li complex with energy only 6 kJ/mol higher than the shown in Fig. 4 – front and top view.

Table S6: BLA and averaged bond lengths in the lithiated [Ni(II)L2]$_n$

n(Li)	BLA	C-COO	CO_q	CO_carb	Ni_O
0Li	0.130	1.477	1.232	1.288	1.897
2Li	0.028	1.452	1.305	1.310	1.908
4Li	0.025	1.440	1.312	1.316	2.192
6Li	-0.031	1.431	1.323	1.342	2.211
8Li	-0.035	1.420	1.340	1.345	2.302
10Li	-0.041	1.411	1.348	1.349	2.345

Table S7: BLA and averaged bond lengths in the lithiated [Ni(IV)L2]$_n$

n(Li)	BLA	C-COO	CO_q	CO_carb	Ni_O	Ni_Cl
0Li	0.134	1.479	1.230	1.281	1.936	2.210
2Li	0.066	1.463	1.281	1.282	1.946	2.209
4Li	0.063	1.448	1.296	1.289	2.015	2.218
6Li	0.022	1.431	1.303	1.301	2.033	3.495
8Li	-0.034	1.422	1.328	1.343	2.187	3.614
10Li	-0.046	1.406	1.338	1.350	2.317	3.687

Table S8: Averaged AIM charges in the lithiated $[Ni(II)L2]_n$

n(Li)	C_ring	O_quin	O_carbox	Ni	Li
0Li	0.519	-1.136	-1.078	1.020	
2Li	0.245	-1.311	-1.201	0.925	0.909
4Li	0.175	-1.476	-1.319	0.730	0.898
6Li	0.066	-1.519	-1.504	0.307	0.887
8Li	-0.112	-1.640	-1.533	0.278	0.796
10Li	-0.135	-1.684	-1.540	0.258	0.731

Table S9: Averaged AIM charges in the lithiated $[Ni(IV)L2]_n$

n(Li)	C_ring	O_quin	O_carb	Cl	Ni	Li
0Li	0.481	-1.117	-1.052	-0.418	1.246	
2Li	0.380	-1.249	-1.156	-0.494	1.155	0.918
4Li	0.350	-1.264	-1.218	-0.634	0.856	0.904
6Li	0.327	-1.277	-1.261	-0.751	0.529	0.898
8Li	0.315	-1.448	-1.364	-0.818	0.400	0.852
10Li	0.199	-1.514	-1.593	-0.864	0.307	0.838

Table S10: BLA and averaged bond lengths in the lithiated $[Ni(II)L3]_n$

n(Li)	BLA	C-COO	CO_q	CO_carb	Ni_O	C-CN	CN
0Li	0.130	1.489	1.226	1.290	1.906	1.422	1.169
2Li	0.027	1.467	1.280	1.307	1.919	1.419	1.171
4Li	-0.015	1.422	1.295	1.317	2.029	1.404	1.186
6Li	-0.043	1.404	1.321	1.325	2.037	1.400	1.204
8Li	-0.055	1.396	1.332	1.331	2.083	1.383	1.257
10Li	-0.066	1.376	1.347	1.336	2.550	1.397	1.295

Table S11: BLA and averaged bond lengths in the lithiated $[Ni(IV)L3]_n$

n(Li)	BLA	C-COO	CO_q	CO_carb	Ni_O	Ni_Cl	C-CN	C-N
0Li	0.207	1.481	1.224	1.273	1.937	2.206	1.430	1.163
2Li	0.072	1.477	1.267	1.278	2.065	2.232	1.423	1.168
4Li	0.022	1.456	1.276	1.294	2.125	3.455	1.416	1.173
6Li	-0.022	1.424	1.327	1.316	2.266	4.063	1.391	1.183
8Li	-0.045	1.411	1.346	1.347	2.366	4.240	1.405	1.195
10Li								

Table S12: Averaged AIM charges in the lithiated $[Ni(II)L3]_n$

n(Li)	C_ring	O_carb	O_quin	N	Ni	Li
0Li	0.630	-1.031	-0.988	-0.957	1.010	
2Li	0.547	-1.133	-1.348	-1.124	0.931	0.918
4Li	0.470	-1.211	-1.388	-1.274	0.731	0.907
6Li	0.404	-1.277	-1.486	-1.324	0.626	0.882
8Li	0.351	-1.378	-1.587	-1.389	0.400	0.853
10Li	0.254	-1.398	-1.641	-1.480	0.290	0.840

Table S13: Averaged AIM charges in the lithiated $[Ni(IV)L3]_n$

n(Li)	C_ring	O_carb	O_quin	Cl	N	Ni	Li
0Li	0.547	-0.987	-0.922	-0.411	-1.142	1.278	
2Li	0.461	-1.065	-1.126	-0.530	-1.259	1.129	0.909
4Li	0.440	-1.168	-1.302	-0.814	-1.406	1.041	0.903
6Li	0.402	-1.232	-1.365	-0.898	-1.538	0.623	0.925
8Li	0.373	-1.542	-1.487	-0.917	-1.581	0.780	0.896
10Li	0.257	-1.750	-1.614	-0.936	-1.627	0.422	0.859

Table S14: Comparison between experimental and calculated electrochemical potentials (at the BLYP/6-311++G** level of theory) of quinone-based molecules vs. Li^+/Li^0 in the solid and the gas phase.

Compound	E^0_{sol}, V	E^0_{gas}, V	E_{exp}, V	$E_{gas} - E_{exp}, V$
p-benzoquinone[49]	0.87	2.18	2.75	-0.57
Anthraquinone[76]	0.43	1.74	2.30	-0.56
2-Hydroxy-1,4-naphthoquinone[76]	0.86	2.17	2.40	-0.23
2,3-Diamino-1,4-naphthoquinone[77]	0.56	1.87	2.25	-0.38
1,1'-Iminodianthroquinone[76,a]	0.44	1.76	2.05	-0.29
1,4-benzoquinone dimer[78]	1.52	2.84	3.00	-0.16
1H-naphtho[2,3-d]imidazole-4,9-dione[77]	0.85	2.16	2.44	-0.28
Lithium azobenzene-4,4'-dicarboxylate[76]	0.36	1.67	1.50	0.17

[a] Calculations are performed on 1-aminoanthraquinone to reduce computational effort

References as cited in the main text:

49. Kwon, J.E.; Hyun, C.-S.; Ryu, Y.J.; Lee, J.; Min, D.J.; Park, M.J.; An, B.-K.; Park, S.Y. Triptycene-Based Quinone Molecules Showing Multi-Electron Redox Reactions for Large Capacity and High Energy Organic Cathode Materials in Li-Ion Batteries. *J. Mater. Chem. A* **2018**, *6*, 3134–3140. https://doi.org/10.1039/C7TA09968A.

76. Xu, D.; Liang, M.; Qi, S.; Sun, W.; Lv, L.-P.; Du, F.-H.; Wang, B.; Chen, S; Wang, Y.; Yu, Y. The Progress and Prospect of Tunable Organic Molecules for Organic Lithium-Ion Batteries. *ACS Nano* **2021**, *15*, 47−80.

77. Lee, J.; Kim, H.; Park, M.J.; Long-life, high-rate lithium-organic batteries based on naphthoquinone derivatives. *Chem. Mater.* **2016**, *28*, 2408−2416.

78. Yao, Z.; Tang, W.; Wang, X.; Wang, C.; Yang, C.; Fan, C.; Synthesis of 1,4-benzoquinone dimer as a high-capacity (501 mAhg−1) and high-energy-density (> 1000 Whkg−1) organic cathode for organic Li-Ion full batteries. *J. Power Sources* **2020**, *448*, 227456.
